# Towards the Development of AgoKirs: New Pharmacological Activators to Study K_ir_2.x Channel and Target Cardiac Disease

**DOI:** 10.3390/ijms21165746

**Published:** 2020-08-11

**Authors:** Laura van der Schoor, Emma J. van Hattum, Sophie M. de Wilde, Netanja I. Harlianto, Aart-Jan van Weert, Meye Bloothooft, Marcel A. G. van der Heyden

**Affiliations:** 1 Honours Program CRU+ Bachelor, University Medical Center Utrecht, Heidelberglaan 100, 3584 CM Utrecht, The Netherlands; l.vanderschoor@students.uu.nl (L.v.d.S.); e.j.vanhattum@students.uu.nl (E.J.v.H.); s.m.dewilde@students.uu.nl (S.M.d.W.); n.i.harlianto@students.uu.nl (N.I.H.); a.vanweert@students.uu.nl (A.-J.v.W.); 2 Department of Medical Physiology, Division of Heart & Lungs, University Medical Center Utrecht, Yalelaan 50, 3584 CM Utrecht, The Netherlands; M.Bloothooft-3@umcutrecht.nl

**Keywords:** inward rectifier channel, K_ir_2, agonist, I_K1_, Andersen syndrome, heart failure

## Abstract

Inward rectifier potassium ion channels (I_K1_-channels) of the K_ir_2.x family are responsible for maintaining a stable negative resting membrane potential in excitable cells, but also play a role in processes of non-excitable tissues, such as bone development. I_K1_-channel loss-of-function, either congenital or acquired, has been associated with cardiac disease. Currently, basic research and specific treatment are hindered by the absence of specific and efficient K_ir_2.x channel activators. However, twelve different compounds, including approved drugs, show off-target I_K1_ activation. Therefore, these compounds contain valuable information towards the development of agonists of K_ir_ channels, AgoKirs. We reviewed the mechanism of I_K1_ channel activation of these compounds, which can be classified as direct or indirect activators. Subsequently, we examined the most viable starting points for rationalized drug development and possible safety concerns with emphasis on cardiac and skeletal muscle adverse effects of AgoKirs. Finally, the potential value of AgoKirs is discussed in view of the current clinical applications of potentiators and activators in cystic fibrosis therapy.

## 1. K_ir_2.x Expression, Structure and Rectification

K_ir_2.x potassium channels are part of the inward rectifier K^+^ (I_K1_) channels family, which are of major importance for stabilizing the resting membrane potential of excitable cells and contribute to final action potential repolarization in cardiomyocytes [1,2]. Additionally, I_K1_ has an important role in non-excitable cells, for example, in osteoblasts, in which I_K1_ channels are involved in chondrogenesis and osteoblastogenesis [3].

K_ir_2.x channels are expressed in several excitable tissues, such as skeletal muscle (K_ir_2.1, K_ir_2.2, and K_ir_2.6), brain (K_ir_2.1, K_ir_2.2, and K_ir_2.3), and heart (K_ir_2.1, K_ir_2.2, and K_ir_2.3) [4]. Within tissues, isoform expression can vary. For example, K_ir_2.3 is dominantly expressed in the atria, whereas K_ir_2.1 is mainly present in the ventricles and has a higher channel density [5]. An intermediate level of expression of K_ir_2.x channels is present in smooth muscle tissue and the retina [4].

I_K1_ channels are homo- or heterotetrameric assemblies of K_ir_2.x monomeric subunits [6,7]. An individual subunit consists of a transmembrane and a cytoplasmic region. The transmembrane region of the channel controls ion selectivity and channel gating, and the cytoplasmic domain functions as a gating regulator [1].

Depending on the membrane potential (V_m_) relative to the potassium equilibrium potential (E_k_), I_K1_ channels conduct either inward or outward current. The outward currents are smaller due to rectification properties. Inward rectification is based on binding of polyamines and Mg^2+^ in the conducting pore region, at V_m_ more positive than E_k_ and inhibiting outward current [8]. Polyamine and Mg^2+^ dependent blocks are absent at more negative V_m_ than E_k_, which results in inward current [9]. Each K_ir_2.x isoform displays its own characteristic rectification profile [5].

Whereas much knowledge on K_ir_2.x function has been derived from experimental in vitro studies and transgenic mice, insights on the physiological contributions of I_K1_ in larger organisms can only be deduced from a few experimental large animal studies using specific inhibitors [10,11] and patients with gain- or loss-of-function mutations [5]. For example, specific I_K1_ inhibition in awake dogs induced adverse effects like premature ventricular contractions, respiratory distress, and mild generalized muscle weakness [11]. On the contrary, the effects of specific I_K1_ activation have not been investigated in large animals. Therefore, as argued before [12], specific I_K1_ modifying compounds, i.e., inhibitors and activators, would benefit experimental studies on the role of I_K1_ in muscle contraction, cardiac function, neuronal excitation, and bone development.

## 2. K_ir_2.x Disease Relationships

I_K1_ function and disease have a bidirectional relationship. On the one hand, mutations can affect K_ir_2.x function, while, on the other hand, diseased states influence K_ir_2.x function. Both congenital and acquired loss-of-function may result from impaired functional K_ir_2.x expression at the plasma membrane, for example, due to aberrant transcription, trafficking, or gating kinetics.

Gain-of-function mutations in the *KCNJ2* gene encoding K_ir_2.1 are associated with ventricular arrhythmia like short QT syndrome type 3 [13] or atrial arrhythmia such as congenital atrial fibrillation (AF) [14,15]. Loss-of-function mutations in *KCNJ2* are associated with autosomal dominant Andersen syndrome (AS) [16]. This disease is characterized by symptoms like ventricular arrhythmias, periodic muscle paralysis, and dysmorphologies such as a broad forehead, a cleft palate, and small hands and feet [17].

AF results in increased I_K1_ densities in atrial tissue [18,19,20], which thereafter causes a shortening of the atrial effective refractory period that ultimately promotes and stabilizes atrial re-entry. Chronic heart failure (HF) induces loss-of-function of I_K1_. In experimental models of HF, I_K1_ is reduced in canine and rabbit ventricles [21,22,23]. More recently, heart failure following myocardial infarction (MI) in a porcine model shows significantly decreased I_K1_ density [24]. Also, declined density of whole-cell I_K1_ was also found in terminal HF patients [25].

**Figure 1:**
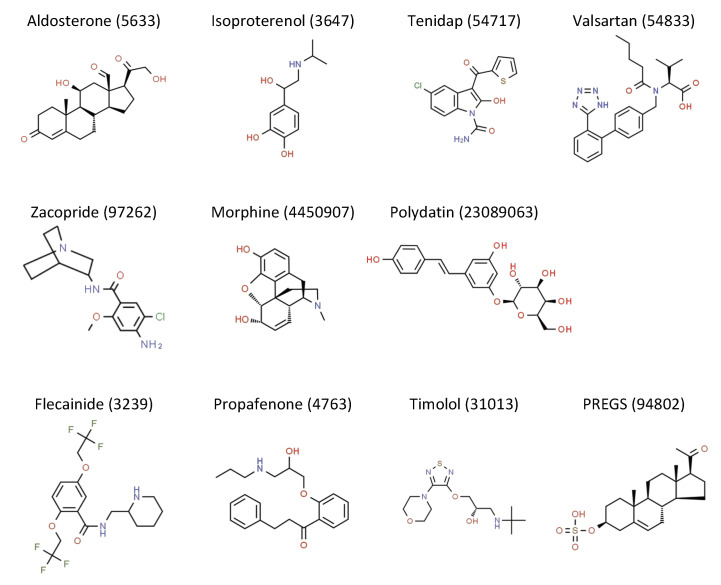
Chemical structures of I_K1_ activating compounds, obtained from the Royal Society of Chemistry-owned ChemSpider website (accessed on 12 June 2020). Structure name and ChemSpider ID is given. Full records can be retrieved at http://www.chemspider.com/Chemical-Structure.ID.html, in which ID should be substituted by the ID number provided above the structure.

Inward rectifier current inhibition has been suggested as a potential avenue in AF therapy [26]. Indeed, in preclinical and clinical settings, pharmacological I_K1_ inhibition is an effective method of AF reduction [10,27]. Moreover, pharmacological activators have been developed for several ion channels and successfully implemented in clinical therapy to treat cystic fibrosis, hypertension, and alopecia, to name a few applications [28,29,30]. Currently, no drugs are available that specifically target impaired I_K1_, whereas such a treatment may be effective in AS and HF. However, twelve drugs and compounds exert I_K1_ activating capacity (Figure 1, Table 1). We will review these drugs for indirect and direct properties to activate the different K_ir_2.x channels, focusing on K_ir_2.1, K_ir_2.2, and K_ir_2.3.

## 3. AgoKirs, Agonists of K_ir_2.x Function

### 3.1. Indirect Activators

#### 3.1.1. Aldosterone

Aldosterone is part of the renin-angiotensin-aldosterone-system (RAAS) and is essential in Na^+^ homeostasis. Activation of aldosterone and the mineralocorticoïdreceptor (MR) play an important role in the pathophysiology of cardiovascular disease.

In vitro studies using isolated rabbit ventricular cardiomyocytes demonstrated that 10 nmol/L of aldosterone caused a rapid activation (within minutes to days) of a 30 pS K^+^-selective current.

This current had pharmacological and biophysical properties that are consistent with those of the I_K1_-current. By use of RU28318, a specific MR-antagonist, and potassium canrenoate, a non-specific MR-antagonist, the underlying mechanism was determined as independent of the MR pathway [34].

Furthermore, cardiac tissue of rats implanted with osmotic minipumps showed a dose-dependent aldosterone effect on K_ir_2.1 expression after four weeks. At a dose of 0.5 µg/h, a moderate increase of K_ir_2.1 and K_ir_2.3 was observed [35], whereas, in another study, decreased expression levels of K_ir_2.1 surprisingly was shown at 1 µg/h aldosterone [45].

#### 3.1.2. Isoproterenol

Isoproterenol, or isoprenaline (ISO), is a synthetic β-adrenoceptor (β-AR) agonist that has a cardiac muscle stimulating and bronchodilating effect. β3-AR stimulation leads to intracellular signal transduction pathways, including protein kinase A and C (PKA and PKC) [46]. In *Xenopus* oocytes, 10 µM ISO activated K_ir_2.2 currents via a PKA-dependent pathway [36], consistent with findings on the role of PKA in K_ir_2.2 activation [47]. Furthermore, ISO also activated K_ir_2.1 currents via a PKC-dependent pathway [36].

#### 3.1.3. Tenidap

Tenidap is a non-steroidal cyclooxygenase and lipoxygenase inhibitor [48] but also a potent K_ir_2.3 opener. By use of ^86^Rb^+^ cell efflux and patch clamp electrophysiology, it was found that tenidap increased K_ir_2.3 carried inward and outward current in Chinese hamster ovary (CHO) cells [44]. Tenidap showed some channel specificity as it did not enhance K_ir_2.1 and K_v_1.5 channel activity [44]; however, it did activate I_KATP_ channels [49].

#### 3.1.4. Valsartan 

Valsartan is a highly selective angiotensin type 1 receptor antagonist and is widely used in the treatment of mild to moderate essential hypertension [50]. In rats with MI, induced by coronary artery ligation, K_ir_2.1 mRNA, protein, and the resulting I_K1_ became downregulated, which was associated with ventricular arrhythmias [51]. Such downregulation of K_ir_2.1/I_K1_ was prevented by Valsartan [37,38,39], possibly involving multiple mechanisms.

Firstly, casein kinase 2 dependent K_ir_2.1 downregulation after MI was prevented by valsartan. Secondly, valsartan treatment after MI decreased T-helper cell levels and thereby ameliorated I_K1_/K_ir_2.1 downregulation. Thirdly, valsartan reduced miRNA-16 levels by the prevention of NF-κB upregulation and thereby prevented downregulation of K_ir_2.1/*KCNJ2*/I_K1_ in infarcted hearts. Currently, it is unknown to what extent the working mechanism of valsartan on each individual signaling pathway contributed to restoring normal K_ir_2.1 expression in MI rat hearts. Nevertheless, the authors stated that a direct interaction of valsartan with the K_ir_2.1 ion channel, resulting in its activation, appeared improbable.

#### 3.1.5. Zacopride

Zacopride (ZAC) is an antiemetic, gastroprokinetic, and anxiolytic drug. It is a selective antagonist of the 5-hydroxytryptamine (5-HT)3 receptor and agonist of 5-HT4 receptor. In the adrenal glands, ZAC is known to stimulate the secretion of aldosterone [52].

Rats treated with ZAC showed elevated levels of K_ir_2.1 protein in left ventricular tissue [53]. Furthermore, ZAC treatment prevented ischemia-mediated downregulation of left ventricular K_ir_2.1 protein [54] (note [55]). Additionally, ZAC enhanced both the inward and outward I_K1_ current in rat ventricular myocytes [56,57] but not in atrial cardiac myocytes [40]. ZAC-induced K_ir_2.1 channel activation appeared to be mediated by PKA-dependent phosphorylation of Ser425 in the K_ir_2.1 C-terminus [40]. In human embryonic kidney 293 cells and CHO cells, ZAC increased I_K1_ carried by ectopic homotetrameric K_ir_2.1 channels but not current carried by homotetrameric K_ir_2.2 or K_ir_2.3 channels or heterotetrameric channels containing K_ir_2.1, K_ir_2.2, or K_ir_2.3 [40,57].

### 3.2. Direct Activators

Within the class of direct activators, interactions with the extracellular domain and cytoplasmic domain have been indicated. The direct activators currently described display isoform specificity and may open the way towards tissue-specific activation of I_K1_.

#### 3.2.1. Flecainide

Flecainide, a class Ic antiarrhythmic drug, is known to block Na^+^ channels and voltage-dependent K^+^ (K_v_) channels. Thereby, flecainide effectively prolongs action potential duration (APD) in the atria but not in the ventricles [58]. On the other hand, flecainide increases I_K1_ selectively in the ventricles, offering a possible explanation for a difference in effect on atrial and ventricular APD [31].

Flecainide specifically activated K_ir_2.1 channels by a mechanism involving Cysteine311 (Cys311) and had no effect on K_ir_2.2 or K_ir_2.3 channels that contain an alanine instead of a cysteine residue on their equivalent positions (312 and 303, respectively) [31].

Flecainide’s mode of action likely involved antagonizing spermine-mediated rectification, resulting in increased outward current. Spermine was shown to inhibit I_K1_ in a concentration-dependent manner. The presence of flecainide decreased spermine’s inhibiting effect, as a rightward shift in the concentration-effect curve was observed [31]. The fact that the E_max_ of spermine was saturated at 82.1 ± 5.5% in the presence of flecainide suggests that spermine block was decreased in a noncompetitive manner by flecainide through allosteric changes to the binding site for polyamines [31].

#### 3.2.2. Propafenone

Propafenone, just like flecainide, is a class Ic antiarrhythmic drug, mainly used in the treatment of ventricular tachycardias. Supra-therapeutic concentrations (>1 µM) of propafenone inhibited K_ir_2.x current [59]. Propafenone interacted with the cytoplasmic domain of the channel, which decreased the negative charge of the pore and the channel affinity for PIP_2_, a lipid critical for channel activation [59].

Given its structural similarities with flecainide, the effect of propafenone on K_ir_2.1 carried current was tested at lower concentrations. At 0.5 μM, propafenone enlarged inward and outward K_ir_2.1 current [33]. Propafenone significantly decreased spermine-induced block and thus relieved inward rectification, similarly as observed for flecainide [33]. However, propafenone failed to enhance both inward and outward K_ir_2.2 and K_ir_2.3 carried current [33]. In cells expressing K_ir_2.1/2.2, K_ir_2.1/2.3, and K_ir_2.2/2.3 heterotetrameric channels, propafenone also failed to increase inward and outward current [33]. Propafenone also did not modify I_K1_ recorded in human right atrial myocytes [33].

The molecular structure of propafenone can be described as an L-like shape, in which a long and short arm are joined by an aromatic ring [33]. Molecular dynamics simulations predicted interaction with K_ir_2.1, in which the hydrophobic long arm of propafenone embeds in a hydrophobic pocket formed by subunit A of the channel. The short arm, on the other hand, contains a group that formed a hydrogen bond with cysteine residue Cys311, which is part of the G-loop [33,60]. This critical hydrogen bond between Cys311 and propafenone was promoted due to the binding orientation of the alkylamino tail, which enabled propafenone’s hydroxyl group to be in close proximity to Cys311 [33].

To determine a potential role of Cys311 and propafenone interaction, mutations creating a cysteine were made in K_ir_2.2 (A312C) and K_ir_2.3 (A303C). These mutations made the channels responsive to activation with 0.5 µM propafenone. With respect to channel kinetics, it was found that opening frequency and mean open probability were increased at all tested voltages [33].

#### 3.2.3. Timolol

Structural similarities and the common Cys311 binding site of flecainide and propafenone have led to the development of a pharmacophore model for binding to this particular site. The model predicted that a drug must have the following molecular properties that enable interaction with Cys311: (1) an “L-like” configuration with a short and long arm, joined by an aromatic ring at an angle of approximately 100 degrees; (2) a hydrogen bond acceptor/donor group in the short arm that interacts with Cys311; (3) a hydrophobic group in the long arm that interacts with a hydrophobic pocket in subunit A of K_ir_2.1; (4) a hydrogen bond between Arg67 or Glu63 and the drug for stabilization. By screening drugs for these properties, timolol was found as a potential activator of K_ir_2.1 [33]. Timolol, a non-selective β-receptor antagonist, selectively activated K_ir_2.1 by directly binding to Cys311 and thereby increased I_K1_ in the ventricles [33].

#### 3.2.4. Pregnenolone Sulfate

Pregnenolone sulfate (PREGS) is an endogenous neurosteroid. PREGS modulates the function of multiple neurotransmitter receptors and channels, among which were voltage-gated K^+^ channels [61]. PREGS-enhanced K_ir_2.3 carried current in *Xenopus* oocytes when applied extracellularly only, whereas no current response in K_ir_1.1, K_ir_2.1, K_ir_2.2, or K_ir_3.1/K_ir_3.2 channels was observed [32].

### 3.3. Unknown Mechanism of Activation

#### 3.3.1. LPS (lipopolysaccharides)

Lipopolysaccharides (LPS) are an important constituent of the cell wall of gram-negative bacteria. LPS can injure pulmonary microvascular walls. In those vessels, K_ir_2.1 plays a role in vasodilation by modulating the membrane potential and intracellular Ca^2+^ concentration. Treatment of mouse pulmonary microvascular endothelial cells with LPS enhanced K_ir_2.1 channel expression and Ba^2+^ sensitive I_K1_ current [41].

#### 3.3.2. Morphine

Morphine is the main element of opium and is used as an anesthetic or sedative as agonist of the μ, δ, and κ opioid receptors [62]. In rabbit ventricular myocytes, morphine significantly increased I_K1_, independent of the opioid-receptor pathway [42]. In human atrial myocytes, morphine was unable to enlarge I_K1_ [63]. 

#### 3.3.3. Polydatin

Polydatin (PD), also known as piceid (3,4′,5-trihydroxystilbene-3-β-d-glucoside), is a monocrystalline compound found, for example, in *Polygonum cuspidatum* Sieb. et Zucc. (Polygonaceae), peanuts, and grapes. PD has a therapeutic effect on hypertension, arrhythmia, hypertrophy, cardiac ischemia, and heart failure by means of manipulation of Ca^2+^ mobilization [64]. In rat ventricular myocytes, PD increased I_K1_ in a concentration-independent manner [43]. 

## 4. Lead Compounds and Clinical Perspective

Due to their similar chemical structure, the drugs flecainide, propafenone, and timolol are able to directly activate K_ir_2.1 channels by means of their interaction with cysteine residue Cys311. This off-target effect can be exploited for rationalized drug development towards specific I_K1_ activators. This provides a promising perspective in the search for a suitable AgoKir.

As mentioned above, timolol has been found to be an activator of K_ir_2.1 after being selected based on the criteria proposed by a pharmacophore model developed by [33]. This opens the possibility to develop a new drug with a higher specificity for K_ir_2.1 channels that meets the requirements of the model. Medicinal chemistry approaches involving modifications to the long and short arm of one of these three existing drugs might be an important step to explore properties that increase specificity for the K_ir_2.1 channel. For example, flecainide’s principal mechanism of action is the inhibition of cardiac Na_v_1.5 sodium channels [65]. It might be possible to modify parts of the flecainide molecule that, as per the pharmacophore model, are not essential for binding to K_ir_2.1, but are important for interaction with Na_v_1.5 to steer specificity. 

Out of all the drugs reviewed in this article, the ones directly activating K_ir_2.1 by binding to Cys311 provide the most viable basis for the development of AgoKirs. As discussed earlier, I_K1_ channels are homo- or heterotetrameric assemblies of K_ir_2.x monomeric subunits. Only the K_ir_2.1 subunit has the cysteine residue Cys311 as part of its G-loop. K_ir_2.2 and K_ir_2.3 subunits contain an alanine instead of a cysteine residue on their equivalent positions (312 and 303, respectively) [31]. Propafenone and flecainide both cannot activate homo- or heterotetramers of K_ir_2.2 and K_ir_2.3 channels. These observations indicate the potential for isoform-specific K_ir_2.x activating compound development by targeting this channel domain but also indicate that other regions of the channel protein should be targeted for the development of multi-isoform activators. Furthermore, high throughput screening methods, such as automated patch-clamp, optical membrane potential detection, and ion-flux measurements, are being developed that will further aid the generation of AgoKirs [66].

The reports on aldosterone and valsartan on K_ir_2.x/I_K1_ regulation/activation are contradictory. Aldosterone production is part of RAAS, whereas valsartan is an antagonist of that system. Therefore, one would expect that those compounds have opposite effects on the activation and expression of the K_ir_2.1 subunits. The findings, however, showed that valsartan was able to ameliorate MI-induced K_ir_2.1 downregulation. Unfortunately, the results for aldosterone were inconclusive. Two studies demonstrated upregulation, and only one study proved downregulation and therefore matched our expectations. This inconsistency between the outcomes of aldosterone and valsartan might indicate that both compounds use additional, RAAS independent pathways in order to activate I_K1_.

Specific AgoKirs may come with potential adverse effects. Gain-of-function mutations indicate the potential of developing reentry-based arrhythmias, either atrial, ventricular, or both. The underlying mechanism is likely AP shortening and reduction in the effective refractory period. Furthermore, due to the strong stabilization of the resting membrane potential, muscle and neuronal cells harboring I_K1_ may become less or even unexcitable. Therefore, it would be beneficial to develop (1) K_ir_2 isoform-specific AgoKirs and (2) AgoKirs with a wide therapeutic range.

Ion channel activators have been pursued in several other research fields, and, for some, they entered clinical practice. The research field advanced furthest with respect to channel activation is undoubtedly that of cystic fibrosis. This disease results mainly from insufficient cystic fibrosis transmembrane conductance regulator (CFTR) channel activity at the plasma membrane of glandular epithelial cells of, for example, the lungs, sweat glands, and gastrointestinal tract [29]. The CFTR channel research field developed both potentiators and correctors. Potentiators act on plasma membrane-localized channels and increase their open probability, whereas correctors address the impaired trafficking deficiencies and enhance forward trafficking of the channels by approaching several different steps of the trafficking machinery [29]. Clinically, a combination of potentiators and correctors appeared most effective (e.g., [67,68]). With respect to cardiac potassium channel activators, progress has been made for K_v_11.1/I_Kr_. Delayed rectifier I_Kr_ loss-of-function is associated with long QT syndrome type 2 and cardiac arrhythmia [69]. A number of compounds have been developed and tested in animal models for antiarrhythmic properties [70,71,72,73]. Currently, these activators mainly function to enhance channel kinetics resulting in increased potassium current. However, many of the forms of I_Kr_ loss-of-function are due to defective forward trafficking of the K_v_11.1 proteins [74,75,76], and the development of compounds directly addressing this issue may be favorable. Unfortunately, existing enhancers of forward trafficking are also strong K_v_11.1 inhibitors [77]. However, molecular insights in the rescue mechanism, including drug-channel interactions, may result in new rescuers of trafficking that do not display inhibition effects. In addition, activators alone might be sufficient in several conditions to increase current to sufficient levels to counteract arrhythmia [78]. With respect to inward rectifier currents, advances have been made in the application of drugs targeting I_KATP_ channels. For example, minoxidil, an I_KATP_ channel opener, is used as a vasodilator in the treatment of resistant hypertension [28] and to promote hair growth in androgenetic alopecia patients [30]. To our knowledge, specific AgoKirs have not been developed, but giving the new insights from the direct channel activators (flecainide, propafenone, timolol, PREGS) and the successes in other research fields, this development will be a valid way to counteract disease-associated loss-of-function of I_K1_ channels.

## Figures and Tables

**Table 1:** Compounds with I_K1_ activating properties.

Compound	Readout	Test System	Dose-Effect Relation	Mechanism of Action	Ref.
***Direct activators***					
Flecainide	C	CHO cells	I_Kir2.1_ EC_50_/Emax (−50 mV) = 0.4 ± 0.01 μM/53.9 ± 3.6% I_Kir2.1_ EC_50_/Emax (−120 mV) = 0.8 ± 0.01 μM/22.0 ± 1.96%	Interaction with Cys311	[31]
	C	guinea pig ventricular cmc	I_K1_ 1 μM: 19.5 ± 3.2% (−120 mV); 38.0 ± 9.5% (−40 mV)	Interaction with Cys311	[31]
PREGS	C	*Xenopus* oocytes	I_Kir2.3_ EC_50_ (−70 mV) = 15.6 ± 0.9 μM	Binding extracellular site	[32]
Propafenone	C	CHO cells	I_Kir2.1_ EC_50_/Emax (−50 mV) = 12.0 ± 3.0 nM/42.0 ± 2.6%	Interaction with Cys311	[33]
	C	guinea pig ventricular cmc	I_K1_ 0.5 μM: approx 45 ± 5% (−40 mV)	Interaction with Cys311	[33]
Timolol	C	CHO cells	I_Kir2.1_ EC_50_ (−50 mV) = 3.2 ± 0.3 nM	Interaction with Cys311	[33]
***Indirect activators***					
Aldosterone	C	rabbit ventricular cmc	I_K1_ 10 nM: 1.6-fold increase NPo of 30 pS current	MR-independent activation	[34]
		rat ventricle	K_ir_2.1 2.24 mg/h/kg 4 wks: approx. 1.57 ± 0.14 fold K_ir_2.3 2.24 mg/h/kg 4 wks: approx. 1.26 ± 0.15 fold (ns)	Unknown Unknown	[35]
Isoproterenol	C	*Xenopus* oocytes	I_Kir2.1_ EC_50_ = 27.4 nM * I_Kir2.2_ EC_50_ = 17.8 nM *	PKC dependent via β3-AR PKA dependent via β3-AR	[36]
Valsartan	R,P	rat ventricle	10 mg/kg/day for 7 days prevented K_ir_2.1 downregulation	Casein Kinase 2 inhibition and/or Th1 immune response inhibition and/or NF-κB-miR-16 pathway	[37,38,39]
	R,P	ventricular cmc, H9c2 cells	20 μM (48 h) prevented K_ir_2.1 downregulation	Casein Kinase 2 inhibition and/or Th1 immune response inhibition and/or NF-κB-miR-16 pathway	[37,38,39]
	C	rat ventricular cmc	20 μM (48 h) prevented I_K1_ inward current downregulation	Th1 immune response inhibition	[38]
Zacopride	C	rat atrial cmc, HEK-293 cells	I_Kir2.1_ EC_50_ (−50 mV) = 30.7 nM I_Kir2.1_ (−50 mV) = 40.7 ± 9.7%	PKA dependent	[40]
LPS	P,C	mouse pulmonary micro-vascular endothelial cells	10 ng/mL (up to 24 h): 1.5-fold increase K_ir_2.1 10 ng/mL I_Kir2.1_ (inward/outward) = approx. 1.7-fold	Unknown	[41]
Morphine	C	rabbit ventricular cmc	I_K1_ (−60 mV) = 25 ± 9% (0.1 μM); 32 ± 11% (1 μM)	Opioid-receptor pathway independent	[42]
Polydatin	C	rat ventricular cmc	I_K1_ 10 μM: approx. 40% (−100 mV)	Unknown	[43]
Tenidap	C	CHO cells	I_Kir2.3_ EC_50_ = 1.3 μM *	Extracellularly, unaffected by pA2, PKC, and AA secondary pathways	[44]

C, current; cmc, cardiomyocyte; P, protein; R, mRNA. * Voltages unknown.

## Abbreviations

AF	Atrial Fibrillation
AgoKir	Agonist of Kir channel
AS	Andersen Syndrome
CFTR	Cystic Fibrosis Transmembrane Conductance Regulator
CHO	Chinese Hamster Ovary
E_K_	Potassium equilibrium potential
HEK	Human Embryonal Kidney
HF	Heart Failure
I_K1_	Inward rectifier current
I_KATP_	ATP regulated inward rectifier current
ISO	Isoproterenol/isoprenaline
K_ir_2.x	Isoform x of the inward rectifier protein K_ir_2 family
LPS	Lipopolysacharides
MI	Myocardial Infarction
MR	Mineral corticoid Receptor
PD	Polydatin
PKA	Protein Kinase A
PKC	Protein Kinase C
PREGS	Pregnenolone Sulfate
RAAS	Renin-Angiotensin-Aldosterone-System
V_m_	Membrane potential
ZAC	Zacopride
